# Social Factors Associated with AIDS and SARS

**DOI:** 10.3201/eid1111.050424

**Published:** 2005-11

**Authors:** Don C. Des Jarlais, Jennifer Stuber, Melissa Tracy, Susan Tross, Sandro Galea

**Affiliations:** * Beth Israel Medical Center, New York, New York, USA; † Columbia University, New York, New York, USA; ‡ New York Academy of Medicine, New York, New York, USA

**Keywords:** stigmatization, AIDS, SARS, mental health, dispatch

## Abstract

We conducted a survey of 928 New York City area residents to assess knowledge and worry about AIDS and SARS. Specific sociodemographic groups of persons were more likely to be less informed and more worried about contracting the diseases.

Public reaction to emerging infectious diseases is a critical factor in controlling the diseases. Informed behavior change may be needed to control disease transmission. Negative public reactions, such as stigmatizing persons at risk for the disease, may greatly hamper prevention and treatment efforts (*1**,**2*). The current public health strategy to control emerging infectious diseases includes timely and complete public reporting (*3*). Providing timely and complete information, however, cannot determine public reaction to the information.

In this study, we examined contrasting relationships between sociodemographic characteristics and knowledge and worry about AIDS and severe acute respiratory syndrome (SARS). AIDS may be considered the prototype of an emerging infectious disease. While AIDS has received considerable public attention since the early 1980s, the strong emotions associated with it create the possibility of nonrational information processing. The stigmatization of persons with or at risk for AIDS has persisted despite public information about the disease (*4*). In contrast, SARS emerged quite abruptly in 2002–2003 and received intense public media attention, but the disease was declared contained by the World Health Organization in 2003 (*5*); little public media attention has been paid to SARS since then.

## The Study

Data for this study came from a cohort of adults (>18 years of age) who lived in metropolitan New York City (NYC). The cohort was recruited through a random digit dial telephone survey conducted from March 25 to June 25, 2002. Additional details on the sampling are provided elsewhere (*6**,**7*). The response rate was 56%. This rate is typical for well-conducted telephone surveys (*7*).

A total of 1,832 respondents was interviewed from September 24, 2003, to February 29, 2004, for this study. We first asked if respondents had heard about SARS and AIDS; persons who had heard about the diseases were asked if they had heard "a great deal," "some," or "not much" about the diseases. We also asked respondents if they were "not at all worried," "somewhat worried," or "very worried" about contracting the diseases.

The analyses were weighted to correct potential selection bias related to the number of household telephones, persons in the household, and oversampling. The analyses were also weighted to make the sample demographically similar to the NYC metropolitan area population according to US Census 2000. The institutional review board of the New York Academy of Medicine approved the study.

Table 1 presents the sociodemographic characteristics of respondents and their relationships to self-reported knowledge of AIDS and SARS. In this analysis, we compared characteristics of respondents who reported knowing "nothing" or "not much" and "some" or "a lot" about the diseases. We considered respondents who reported knowing "nothing" or "not much" to be poorly informed. Five percent of the respondents reported being poorly informed about AIDS, and 21% reported being poorly informed about SARS. Table 2 presents the sociodemographic characteristics of the respondents and shows their relationship to worry about contracting AIDS or SARS. In this analysis, we examined characteristics of respondents who reported that they were "very worried" about contracting AIDS or SARS. There were no meaningful difference in the percentage of subjects who reported being "very informed" about each disease or "very worried" about contracting each disease.

**Table 1:** Survey findings of respondents' knowledge about AIDS and severe acute respiratory syndrome (SARS) (N = 928)*

Characteristic	Total, n (% )	Poorly informed,		Poorly informed,	
		AIDS, n (%)	p value	SARS, n (%)	p value
Sex					
Male	402 (45.3)	20 (8.0)	0.016	67 (21.7)	0.716
Female	526 (54.7)	13 (2.5)		102 (20.2)	
Race/ethnicity					
White	579 (54.1)	19 (2.9)	0.054	79 (14.6)	<0.0001
Asian	50 (5.0)	5 (16.3)		5 (20.8)	
Black	133 (18.9)	3 (5.7)		30 (17.5)	
Hispanic	131 (19.5)	3 (6.4)		45 (40.6)	
Other	21 (2.6)	2 (11.9)		8 (36.1)	
Age, y					
>65	147 (11.9)	12 (6.6)		52 (37.8)	
55–64	125 (12.4)	5 (9.6)		24 (24.9)	
45–54	185 (18.2)	0 (0.0)		18 (15.7)	
35–44	215 (20.7)	4 (1.8)		30 (14.7)	
25–34	185 (25.8)	7 (3.5)		29 (19.2)	
18–24	61 (11.0)	4 (16.3)		12 (19.6)	0.006
Educational status					
Graduate work	173 (13.8)	2 (3.3)	0.460	10 (12.8)	<0.0001
College degree	306 (30.0)	7 (4.7)		32 (12.8)	
Some college	172 (21.4)	2 (1.8)		23 (17.4)	
High school/general education diploma	186 (25.2)	14 (8.3)		62 (27.2)	
Less than high school	89 (9.6)	8 (7.0)		41 (48.9)	
Marital status					
Married	409 (52.9)	14 (5.6)	0.907	69 (22.4)	0.028
Divorced/separated/widowed	214 (15.8)	14 (5.2)		56 (28.5)	
Never married/unmarried couple	298 (31.3)	5 (4.1)		43 (14.7)	
Household income at baseline					
>$75,000	262 (33.8)	4 (1.3)	0.062	22 (12.3)	0.002
$40,000–$74,999	217 (27.9)	3 (4.1)		24 (12.7)	
$20,000–$39,999	158 (23.2)	9 (7.0)		42 (28.5)	
<$20,000	130 (15.2)	8 (9.6)		47 (36.8)	
Total	928 (100.0)	33 (5.0)		169 (20.9)	

*Poorly informed, respondents reported knowing "nothing" or "little" about the disease.

**Table 2:** Survey findings about respondents' worry about AIDS and severe acute respiratory syndrome (SARS)*

Characteristic	Total, n = 928 (%)	Very worried,		Very worried	
		AIDS, n = 917 (%)	p value	SARS, n = 863 (%)	p value
Sex					
Male	402 (45.3)	20 (6.3)	0.553	10 (2.0)	0.006
Female	526 (54.7)	25 (5.0)		35 (8.0)	
Race/ethnicity					
White	579 (54.1)	8 (1.3)		13 (2.5)	0.028
Asian	50 (5.0)	2 (3.5)		4 (20.0)	
Black	133 (18.9)	15 (8.4)		11 (7.2)	
Hispanic	131 (19.5)	19 (15.4)		15 (8.3)	
Other	21 (2.6)	0 (0.0)		2 (3.8)	
Age, y					
>65	147 (11.9)	4 (1.2)		10 (5.4)	
55–64	125 (12.4)	3 (1.7)		6 (5.5)	
45–54	185 (18.2)	8 (7.5)		6 (7.4)	
35–44	215 (20.7)	11 (4.5)		10 (5.5)	
25–34	185 (25.8)	16 (11.0)		7 (2.6)	
18–24	61 (11.0)	3 (1.1)	0.006	5 (5.9)	0.723
Educational attainment					
Graduate work	173 (13.8)	1 (1.0)	<0.0001	4 (1.7)	0.250
College degree	306 (30.0)	5 (2.0)		13 (4.9)	
Some college	172 (21.4)	7 (1.4)		11 (7.2)	
High school/general education diploma	186 (25.2)	16 (10.4)		8 (3.6)	
Less than high school	89 (9.6)	14 (18.9)		9 (13.3)	
Marital status					
Married	409 (52.9)	15 (4.4)	0.627	13 (5.5)	0.778
Divorced/separated/widowed	214 (15.8)	10 (6.0)		14 (5.4)	
Never married/unmarried couple	298 (31.1)	19 (6.8)		15 (4.0)	
Household income at baseline					
>$75,000	262 (33.8)	4 (1.6)	<0.001	10 (5.9)	0.197
$40,000–$74,999	217 (27.9)	6 (4.2)		6 (1.6)	
$20,000–$39,999	158 (23.2)	11 (4.7)		10 (6.7)	
<$20,000	130 (15.2)	18 (22.8)		11 (8.2)	
Total	928 (100)	45 (5.6)		45 (5.2)	

*Among those who had heard at least something about AIDS (n = 917) and SARS (n = 863), respectively.

The factors associated with being poorly informed and worried about contracting AIDS and SARS varied; respondents in the lower socioeconomic group were likely less informed and more worried about both of the diseases. Particularly, racial/ethnic minority status, lower formal education, and lower income were associated with being poorly informed and worried.

Being poorly informed about AIDS and being poorly informed about SARS were strongly related. Of respondents who reported being poorly informed about AIDS, 78% reported also being poorly informed about SARS; 18% of the respondents who reported not being poorly informed about AIDS reported being poorly informed about SARS (p<0.001). A strong relationship existed between being very worried about both diseases. Of the respondents who reported being very worried about AIDS, 16% reported also being very worried about SARS; 5% of the respondents who were not very worried about AIDS were very worried about SARS (p = 0.016).

Finally, we examined the relationships between being informed and worried about contracting AIDS/SARS. These analyses were confined to respondents who reported having some information about AIDS/SARS; respondents who reported that they had not heard about the diseases were not asked the follow-up questions. In these respondents, no relationship between having heard and being worried about getting the diseases was shown.

## Conclusions

Given the widespread disparities in health among racial/ethnic and socioeconomic groups in the United States (*8*), that these factors were associated with being less informed and more worried about contracting AIDS or SARS was not surprising. The data presented here, however, are likely not related to access to healthcare services (particularly for SARS) and suggest more fundamental issues in obtaining information and developing realistic concerns about diseases. The high percentage of Spanish-speaking respondents who were poorly informed about AIDS and SARS and very worried about getting SARS suggests possible language and cultural issues in acquiring and processing information.

The data from this study were collected in a major city of an industrialized country and should not be generalized to developing and transitional countries. Nevertheless, if obtaining and evaluating information is adversely affected by factors such as low education level, low income, and ethnic minority status, then properly informing the public may be particularly difficult in developing and transitional countries. The epidemiology of AIDS and SARS has been very different in NYC (>58,097 AIDS cases [9], 9 SARS cases). Despite this difference, strong parallels existed in the relationships of socioeconomic factors to knowledge and worry about both diseases.

The limitations of this study included using single items to measure knowledge and worry about AIDS and SARS and the standard limitations of telephone surveys, e.g., inability to contact households without telephones, moderate refusal rates. However, this study strongly suggests that adequate public knowledge and emotional assessment may be critical to control these diseases.

Our data suggest that socioeconomic class and race/ethnicity factors may help shape public understanding of emerging infectious diseases. Targeted communication to different population subgroups may be required to achieve public understanding of an emerging infectious disease.
